# Combined Use of Microwave Sensing Technologies and Artificial Intelligence for Biomedical Monitoring and Imaging

**DOI:** 10.3390/bios16010067

**Published:** 2026-01-22

**Authors:** Andrea Martínez-Lozano, Alejandro Buitrago-Bernal, Langis Roy, José María Vicente-Samper, Carlos G. Juan

**Affiliations:** 1 Elche Microwave Laboratory Research Group, Department of Materials Science, Optics and Electronic Technology, Miguel Hernández University of Elche, 03202 Elche, Spain; 2 Department of Electrical, Computer, and Software Engineering, Ontario Tech University, Oshawa, ON L1G 0C5, Canada; 3 Electrical & Computer Engineering, Faculty of Engineering, Lakehead University, Thunder Bay, ON P7B 5E1, Canada; 4 Research Group on Data Science, Energy, Infrastructure and Building, Department of Civil Engineering, University of Alicante, 03690 San Vicente del Raspeig, Spain; jm.vicentesamper@ua.es; 5 Neuroengineering Biomedical Research Group, Institute of Bioengineering, Miguel Hernández University of Elche, 03202 Elche, Spain

**Keywords:** artificial intelligence, deep learning, electronic biosensors, machine learning, medical imaging, microwave biosensors

## Abstract

Microwave sensing technology is rapidly advancing and increasingly finding its way into biomedical applications, promising significant improvements for medical care. Concurrently, the rise of artificial intelligence (AI) is enabling significant enhancements in the biomedical domain. Close scrutiny of the recent literature reveals intense activity in both fields, with particularly impactful outcomes deriving from the combined use of advanced microwave techniques and AI for biomedical monitoring. In this review, an up-to-date compilation, from the perspective of the authors, of the most significant works published on these topics in recent years is given, focusing on their integration and current challenges. With the objective of analyzing the current landscape, we survey and compare state-of-the-art biosensors and imaging systems at all healthcare levels, from outpatient contexts to specialized medical equipment and laboratory analysis tools. We also delve into the relevant applications of AI in medicine for processing microwave-derived data. As our core focus, we analyze the synergistic integration of AI in the design of microwave devices and the processing of the acquired data, which have shown notable performances, opening new avenues for compact, affordable, and multi-functional medical devices. We conclude by synthesizing the prevailing technical, algorithmic, and translational challenges that must be addressed to realize this potential.

## 1. Introduction

The current landscape of medicine and biomedical science is undergoing a profound transformation driven by the ever-increasing integration of sophisticated electronic technologies. This pervasive integration manifests across the entire healthcare continuum, fundamentally boosting diagnostic capabilities, therapeutic interventions, research methodologies, and patient management paradigms. The digitalization of health records has established the foundation for interconnected data ecosystems, while advanced diagnosis tools leverage complex electronics for unprecedented precision and early disease detection. Current hardware possibilities range from high-resolution digital imaging equipment and miniaturized wearable biosensors to point-of-care genetic sequencers [1,2]. This technological progression finds a particularly compelling expression in microwave sensing devices, which are garnering significant interest due to their unique combination of non-ionizing safety, deep-tissue penetration, and innate sensitivity to the dielectric properties of biological tissues, crucial factors for differentiating pathological states and monitoring physiological processes [3].

Furthermore, telemedicine platforms, powered by robust communication networks and secure data protocols, are dissolving geographical barriers to specialist access and enabling remote patient monitoring, thereby enhancing healthcare delivery efficiency and reach. This data-centric paradigm creates both an opportunity and a bottleneck for advanced analysis. The resulting proliferation of diverse biomedical data has spurred the development of advanced biosensors and AI-driven analysis tools to manage and interpret this information [4,5,6].

Concurrently, the convergence of microwave sensing systems with computational power, particularly through artificial intelligence (AI) techniques, is revolutionizing the analysis of the now massive biomedical datasets, thereby accelerating key aspects of modern medicine such as drug discovery, personalizing treatment strategies, and predicting patient outcomes with increasing accuracy. In recent years, in addition to the expected progress associated with AI, such as early detection of subtle pathologies through AI-driven medical image analysis or predictive analytics for optimized and individualized interventions, AI has also proved beneficial for improving the development of the microwave tools themselves. We are now witnessing the synergistic integration of hardware and software at all stages of design and implementation, with exceptional promise.

As a result, the significant potential of these technologies has led to intense research activity in the realms of AI techniques and microwave sensing applied to biomedicine, as well as their intersections. Indeed, at the time of writing this manuscript a quick search on Scopus database with “artificial”, “intelligence”, “electronic”, and “medical” as keywords yields 6181 results, from which over 77% are from the last 5 years. This prolific academic production demonstrates the interest and potential of these technologies, the boundaries between them becoming progressively blurred. Some recent reviews on these topics can be found. To name a few, we discovered [3] exclusively focused on microwaves in medicine; Ref. [7] focused on electromagnetic technologies for blood glucose level monitoring; Ref. [8] focused on microwave medical imaging techniques; Ref. [9] focused on different techniques and technologies for developing wearable healthcare devices; or Refs. [10,11], focused on different uses and applications of AI in medicine. However, to the best of our knowledge, no works have been published exploring the specific combination of microwave sensing technology and AI for biomedical monitoring and imaging, despite their evident relationship. This gap is notable because microwave sensing presents unique signal processing challenges (e.g., low signal-to-noise ratio, complex dielectric interactions) where AI techniques can be particularly transformative.

In these circumstances, an updated review of the main literature focused on this intersection seems of high relevance. Not aiming at an extensive compilation of the vast available literature, this narrative review has three core objectives:

(1) To survey and critically compare microwave-based devices for physiological monitoring and imaging (Section 2).
(2) To review AI paradigms suited for analyzing microwave-derived data (Section 3).
(3) To analyze the integration of AI at the system level, from algorithm design to embedded implementation (Section 4).

We conclude by synthesizing the key limitations, challenges, and future directions at this interdisciplinary frontier (Section 5). For clarity, this review focuses specifically on diagnostic monitoring and imaging applications, excluding therapeutic microwave uses and implantable systems.

## 2. Microwave Sensing and Imaging Technology in Medicine

Electronics underpin almost all modern fields, and medicine is no exception. From screening instruments to therapy tools, the advances in medicine have been closely linked to advances in technologies over the last several decades. The landscape of electronic biosensors is broadly divided between general health monitoring [12,13,14] and the management of specific chronic conditions [15], including behavioral and psychiatric disorders [16,17]. For general wellness, dominant technologies include hydrogel-based electrodes for biopotentials such as electrocardiography (ECG) or electroencephalography (EEG) [18], wearable iontronic and triboelectric sensors for blood pressure monitoring [19,20], and optoelectronic systems like photoplethysmography (PPG) for vital signs [21]. These same modalities, along with measures of electrodermal activity (EDA) and heart rate variability (HRV), are leveraged for disease-specific management, from guiding rehabilitation processes [22] to adjusting therapies for behavioral disorders [23,24,25] and screening for depression and anxiety.

While this established ecosystem excels at measuring surface-level electrical, optical, and chemical signals, it provides limited direct insight into the internal dielectric properties of tissues. This fundamental limitation defines a compelling niche for microwave-based sensing, which probes tissue composition and deep-tissue physiology to offer unique, complementary data on conditions like blood composition analysis, deep tissue health or detection of specific cells or nanoparticles, as explored in the following subsections. The unique attributes of microwave sensing, including its contactless operation, sensitivity to deep-tissue hydration and composition, and ability to monitor physiological movements, are creating new paradigms for non-invasive, continuous health monitoring. A comparative summary of representative microwave-based devices and systems discussed in this section is provided in Table 1. It should be noted that a comprehensive review of the most significant works on these topics would be lengthy and unwieldy. Instead, we collect a representative selection of proposals and applications drawing significant interest within the research community.

### 2.1. Biomarkers Monitoring

Biomarkers monitoring, with a general emphasis on wearable and outpatient technologies, represents an intensely active field of research, motivated by the generally low-cost nature of implementations and a wide variety of potential applications. This domain has been bolstered by advancements in fabrication techniques, such as flexible and textile-based electronics [26,27,28]. While extensive reviews cover the broader landscape of wearable biosensors [9,29,30], this section focuses specifically on the emerging potential of microwave-based systems. The unique attributes of microwave sensing, including its contactless operation, sensitivity to deep-tissue hydration and composition, and ability to monitor physiological movements, are creating new paradigms for non-invasive, continuous health monitoring.

During the last decades we have witnessed considerable advancements in the development of sensors and instruments for non-invasive measurement of the concentration or presence of certain solutes or compounds, whether in solutions or blood. These are of interest in the biomedical realm, particularly in the measurement of glucose concentration [31,32]. Such sensors find enormous uptake in the management of diabetes and its complications, since they ideally allow for continuous non-invasive monitoring of patients’ blood glucose level, thereby facilitating the right treatment and actions at opportune times [33].

Based on microwave technology, the fundamentals of these sensors were studied in [34]. Their operating principle relies on the influence of glucose on the dielectric permittivity of the medium. The dielectric permittivity is a characteristic of each material that depends on its chemical composition, and that determines the strength of the interaction of the material with electromagnetic fields over a wide range of frequencies. It has been shown repeatedly that, for liquid solutions containing glucose (such as water solutions or blood), a change in the glucose concentration entails a change in the dielectric permittivity of the solution [35,36,37,38,39]. Consequently, dielectric permittivity sensors, which have non-invasive capabilities, could potentially act as practical glucose concentration sensors. These sensors are usually made with planar microwave resonators, the response of which depends on the dielectric permittivity in their vicinity. Functional devices were thoroughly analyzed from a general point of view in [40,41].

**Figure 1:**
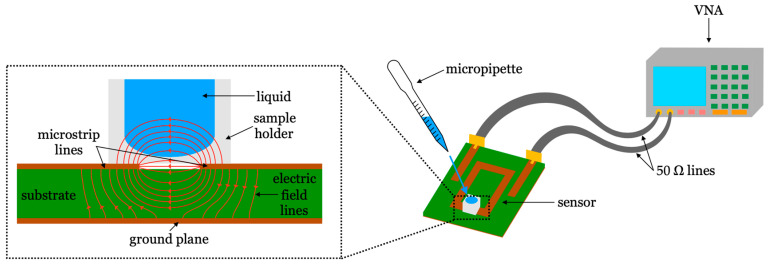
Measurement concept for planar microwave glucose sensors, where the interaction between the electric field lines (red arrows) and the liquid sample (in blue) is seen (VNA = vector network analyzer). Reprinted from [47] under CC license.

Following these principles, numerous works have demonstrated glucose concentration measurement capabilities through different metrics derived from the response of the sensor [42], such as the resonance frequency (*f*_r_) [43], the insertion/return losses [44], the phase of the scattering parameters [45], or the unloaded quality factor (*Q*_u_) [46]. For example, the glucose measurement capabilities of *Q*_u_-based sensors with ternary blood plasma solutions were assessed in [47], a certified biocompatible implementation was proposed in [48], and clinical trials were conducted in [49]. In terms of *f*_r_-based approaches, wireless implementations were proposed in [50,51], a stepped impedance resonator was used in [52], and a dual band split ring resonator was tested in [53] with good resolution. A return loss-based dumbbell defective ground resonator was analyzed in [54], while a plasmonic microwave resonator was assessed with human trials in [55]. The measurement concept of these sensors is illustrated in Figure 1.

In addition to glucose concentration sensing, this technology has been applied to other biosensing scenarios. A planar microwave sensor for the detection of sweat and urine leakage in underwear was studied in [56]. Complementary split-ring resonators were used as blood urea concentration sensors for enhanced dialysis adequacy [57,58]. The denaturation of bovine serum albumin protein, due to the action of urea, was detected through a differential microfluidic sensor based on slot resonators [59]. Lactate monitoring, of interest for hyperlactatemia and aerobic fitness assessment, was achieved in [60,61]. A substrate-integrated waveguide resonator was combined with the lipase enzyme as a bioreceptor for successful estimation of triglycerides mixture in blood serum for heart health monitoring [62]. A sensor based on a split-ring resonator for detecting several types of amino acids for cell cultures and nutrition studies was shown in [63]. Skin hydration was monitored with a resonant sensing tag [64]. Continued progress in microwave biosensors has permitted the accurate detection of nanoparticles [65,66,67], proteins [68], emerging contaminants [69], and bacteria [70,71,72], all of high biomedical interest. Measurement of biosignals and vital signs through microwave devices has also been achieved [50], such as heart beat, in several configurations [73,74], among others [75,76].

Despite the latest advancements, these microwave biosensors will still face several challenges in the near future. Firstly, the high detection standards usually required in medicine translate to considerably high device sensitivities. Ongoing research is starting to yield impressive sensitivity boosting strategies through different approaches, such as coupled-resonator sensors [77,78,79] or so-called lossy sensors [80,81,82], that may lead to unprecedented biosensing capabilities. Secondly, in a complex environment such as the biomedical one, these sensors will have to demonstrate selectivity or specificity high enough for robust, trustable operation, not only against multi-component variations [83], but also against the influence of external parameters such as temperature [84,85] or pressure [86]. This is indeed a challenging issue considering that the effective permittivity is a global parameter for the medium. In this regard, recent progress has been made with differential sensing [87], multi-frequency multi-phenomena sensing [61,88], or even multi-parameter simultaneous sensing [89]. The use of artificial intelligence techniques is expected to have a particularly pronounced impact here, as will be discussed in Section 4. Also, other alternative technologies are under current research, such as glucose measurement from sweat [90,91] by means of an enzymatic graphene sponge [92] or on gel electrolyte [93], for instance. Glucose measurement from saliva has also been investigated [94] by means of optical [95], electrochemical [96], or enzymatic techniques [97,98].

### 2.2. Microbiology- and Cell Biology-Related Equipment

Microbiology and cell biology testing have also been of interest for researchers developing new sensors. A high selectivity can be obtained by capturing single cells using AC dielectrophoresis [99], allowing for the study of intracellular properties. Although cell microscopy using chemical stains or fluorescent dyes to study and sort cells presents several advantages (i.e., high specificity, sensitivity, and efficiency), the required equipment is generally expensive, and the used chemicals may interact with the biomolecules under test, affecting the extracted parameters.

**Figure 2:**
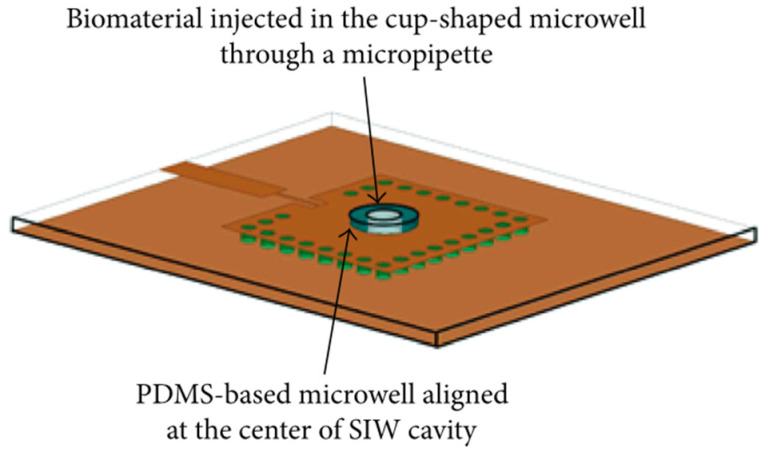
Design of a SIW cavity microfluidic biosensor for fibroblast cells detection while flowing through the cavity. Reprinted from [103] under CC license.

The use of microwave sensing removes the need of chemicals or solutions that can have an impact on the cells under study. Different approaches have been presented over the years, such the integration of microstrip lines and capacitive gaps to determine the dielectric properties of the sample under test [100,101,102]. More recently, significant advances in microwave circuit integration with microfluidics have allowed probing biological samples at microwave and millimeter wave frequencies. For example, in [103], a microfluidic biosensor was implemented using a microwave SIW cavity resonator with a resonant frequency of 13.34 GHz. This sensor can detect fibroblast cells by measuring the frequency shift of the cavity, as shown in Figure 2. Additionally, in [104], a design using an interdigitated capacitor loaded coplanar waveguide was presented to differentiate dead and living cancer cells by determining their dielectric properties at 40 GHz. This integration can even allow scientists to study individual cells as conducted in [105,106] where the principle of label-free cell probing with microwaves in a microfluidic channel is described. A further comparison of microwave and conventional cellular analysis techniques is presented in [107]. The use of these microwave sensors increases the capability of cell differentiation given their morphological similarities.

Other successful examples of integration between microfluidics and microwave sensing strategies make use of coplanar capacitive gaps [108], and coplanar waveguides in frequency ranges between 40 MHz and 40 GHz [109,110,111]. As the frequency of operation of these sensors increase, a higher resolution can be achieved, allowing the characterization of the cell nucleus [112,113]. The successful implementation of microwave sensors allowing cell characterization to a nucleus level allows researchers to better identify and differentiate these cells in details that were not imaginable years ago.

### 2.3. Medical Imaging

Medical imaging equipment consists of non-invasive tools used to visualize the interior of the human body, either for clinical analysis or to detect abnormalities. Traditional imaging methods that provide information about tissues, such as X-rays, computed tomography (CT), magnetic resonance imaging (MRI), or diagnostic echography, among others, have evolved over time, improving their capabilities [114]. However, these methods still present various limitations, including their complexity, high cost due to maintenance and implementation requirements, bulkiness, portability challenges, low sensitivity to tissue changes, and exposure to harmful radiation and nephrotoxic contrast agents in certain cases. On the other hand, they offer the significant advantage of providing high resolution and precision in images, creating a strong demand for medical imaging systems that are safe, reliable, and cost-effective [115,116,117]. In addition to the above-mentioned limitations, in systems based on ionizing radiation, the permissible exposure dose is also limited. Consequently, significant efforts have been devoted to finding a reliable medical imaging technology that utilizes non-ionizing radiation while remaining user-friendly and cost-effective. One such alternative technology for image reconstruction is based on electromagnetic energy, specifically in the microwave frequency range [116].

Microwave imaging (MWI) is a non-invasive and non-destructive technique aimed at detecting materials or targets in order to obtain their physical properties and/or information about the conditions of the structures under study [118]. These techniques typically rely on near-field measurement systems capable of capturing the electromagnetic field resulting from the interaction between incident waves and the materials or targets in question [118]. Medical MWI uses electromagnetic waves, typically in the range of 1 to 10 GHz. The frequency range of these systems is usually selected to ensure appropriate signal penetration depth and spatial resolution [119,120]. MWI is an alternative to traditional imaging methods due to its numerous and significant advantages, such as the use of low-power, non-ionizing signals, making it a low-risk health method that can be safely repeated more frequently compared to conventional imaging systems [121]. Additionally, it is a cost-effective technique, since the equipment used is generally inexpensive and can even be made portable, opening the door to outpatient usage.

However, MWI techniques also present some disadvantages and technical challenges, such as limited spatial resolution [115,116]. Medical MWI systems comprise a hardware component responsible for data acquisition and a software component to process the data. Over the years, research on these medical MWI systems has been motivated by its significant potential in monitoring various pathological conditions [115]. Examples of applications of these systems include breast cancer detection [122,123,124], monitoring of bone fractures [125,126], portable knee imaging systems [127], neck tumor [128] and skin tumor [129] detection, human torso scanners [130], lung cancer detection [124,131], human heartbeat detection systems [132,133], congestive heart failure detection [134], dynamic imaging for cardiovascular systems [135], muscle tear imaging [136], and detection of brain abnormalities such as strokes [137,138], brain tumors [124,139], or intracranial hemorrhages [140,141], among others.

The basic principle of microwave medical imaging is the contrast in tissues due to their different dielectric constants, which is used to reconstruct the backscattered signals and to generate the corresponding images by means of processing algorithms [142]. These algorithms vary depending on whether the system is tomographic or radar-based [115]. Different tissues through which the electromagnetic signals travel exhibit varying dielectric properties. Due to these differences, image reconstruction algorithms can display the tissues the signal travelled through and even locate specific elements within the body, such as malignant or tumor tissues, which tend to scatter signals more intensely than healthy tissues [115]. This is a result of healthy and malignant biological tissues being morphologically distinct, a direct consequence of variations in total cell water content and changes in cell membrane properties [120].

In the literature, three methods of medical MWI systems have been explored, conventionally classified as passive, hybrid, and active methods. The passive method uses a radiometry device to measure temperature differences between healthy and malignant tissues, since malignant tissues exhibit higher temperatures compared to surrounding normal tissues. The hybrid method, also known as the thermoacoustic method, employs microwave sensors and ultrasonic transducers to detect malignant tissues. In this case, tissues are illuminated by electromagnetic waves at microwave frequencies, which are absorbed by the tissue. Malignant tissues absorb more energy than normal tissues and emit stronger acoustic waves due to their higher electrical conductivity compared to surrounding normal tissues. An ultrasonic transducer then measures the reflected acoustic signals and processes them to construct an image. The active method uses microwave signals to illuminate tissues, and the variation in dielectric properties between normal and malignant tissues is measured within a specific microwave frequency range. The backscattered signals from the tissues are estimated to construct an image indicating the presence of malignant tissue based on the reflected signals, which are stronger than those from healthy tissues [143].

**Figure 3:**
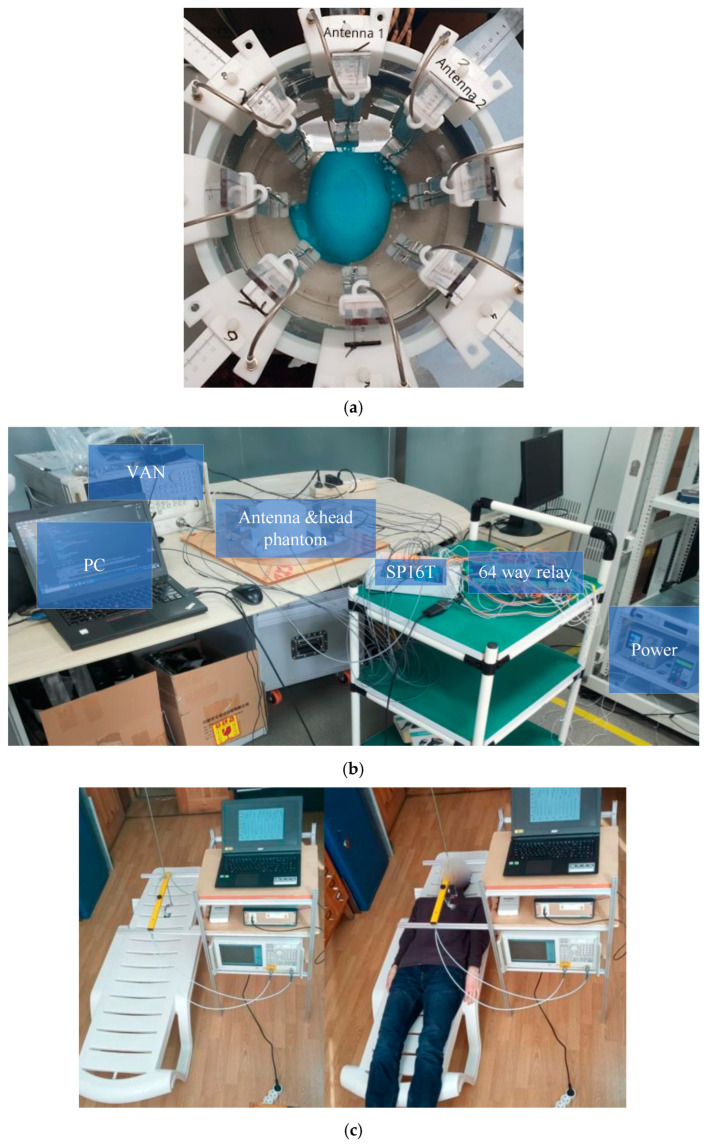
Examples of microwave tomography systems: (**a**) system for cerebrovascular accidents detection, reprinted from [137]; (**b**) brain imaging system, reprinted from [150]; and (**c**) system for assessing lung and cardiovascular activity, reprinted from [152]. All images are reprinted under CC license.

Techniques for microwave imaging using active methods can be divided into two main groups: microwave tomography and radar-based imaging. Microwave tomography applies microwave signals to illuminate the tissue under study and provides quantitative information about the dielectric properties of the tissue to identify malignant tissues. This is achieved by generating a permittivity and conductivity map through the inversion of these signals. This method typically employs iterative image reconstruction algorithms and can be represented as a nonlinear inverse problem, which is time-consuming due to the complexity of the computational process, requiring significant computational resources to obtain a solution. If these inverse scattering approaches are poorly formulated, the results may lack uniqueness, necessitating regularization to achieve convergence toward a meaningful solution. Images of the tissue under study can be constructed from microwave data using inverse scattering algorithms, which estimate the constitutive parameters of tissues by analyzing absorbed and reflected microwave signals [143,144]. Examples of medical imaging systems using microwave tomography include breast cancer detection [145,146,147,148], stroke detection [137,149], brain imaging [150], shoulder injury detection [151], lung and cardiovascular activity measurement [152], and axillary lymph node detection [153], among others. Some of these systems are illustrated in Figure 3.

**Figure 4:**
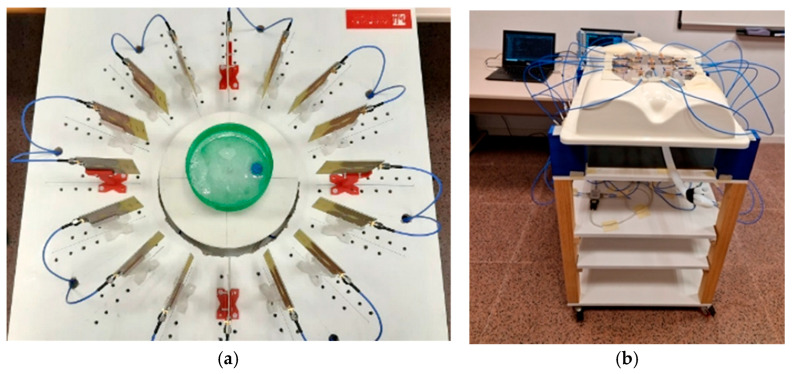
Examples of microwave radar-based medical imaging systems: (**a**) system for breast cancer detection, reprinted from [123]; (**b**) system for detection of abdominal aortic aneurysm, reprinted from [161]. All images are reprinted under CC license.

Regarding radar-based techniques, these ones utilize an external microwave source to illuminate the object of interest with wideband signals. The reflected signals from the object are then used to detect differences in dielectric properties between normal and malignant tissues, enabling the identification and localization of potential malignant tissues [143,154]. The radar-based method, by employing wideband signals, satisfies the resolution requirement while maintaining adequate signal penetration as tissue conductivity increases with frequency. The optimal frequency range for such systems usually lies between 1 and 10 GHz. When the transmitting antenna irradiates short-duration microwave energy bursts toward the object under study, any dielectric discontinuity encountered by the wave will cause a reflection. These reflected signals are captured by the receiving antenna, which estimates the existence, location, and characteristics of the structure causing that discontinuity [154]. This method is both more convenient and safer than microwave tomography [144]. Examples of radar-based medical imaging systems can be found in various monitoring applications, such as breast cancer detection [123,155,156,157], brain-shift detection [158], brain anomaly detection [159,160], knee injury imaging [127], abdominal aortic aneurysm detection [161], bone fracture detection [162], or cardiovascular activity detection [135], to name a few. Two of these systems are illustrated in Figure 4.

**Table 1:** Summary of microwave-based devices and systems for biomedical monitoring and imaging.

Device/System Type	Target Physiological Parameter (s)	Measurement Principle	Key Specifications	Critical Comments/Key Advantages and Limitations
Planar Resonant Glucose Sensors [42,43,44,45,46,47,48,49,50,51,52,53,54,55]	Glucose concentration in blood, plasma, or interstitial fluid.	Complex dielectric permittivity shift measured via resonance frequency (*f*_r_), quality factor (*Q_u_*), or S-parameter magnitude/phase.	1–10 GHz range; planar PCB technology; often integrated with microfluidic channels.	Adv.: Strong potential for non-invasive, continuous monitoring; compatible with low-cost fabrication. Lim.: Fundamental challenge of selectivity in complex bio-fluids; sensitive to confounding variables (temperature, pressure, other solutes). AI and multi-parameter strategies are key research directions [83,89].
Biomarker-Specific Resonant Sensors [56,57,58,59,60,61,62,63,69,70,71,72]	Lactate, urea, albumin, triglycerides, amino acids, skin hydration, bacteria, contaminants.	Change in resonant frequency or S-parameter-derived metrics due to binding or dielectric change from target analyte.	Various frequencies (MHz to GHz); planar resonators (CSRR, SRR); often functionalized or used with microfluidics.	Adv.: High specificity possible with functionalization (e.g., enzymes, bioreceptors); label-free detection. Lim.: Requires careful bioreceptor integration and stability; sensitivity must be extremely high for trace biomarkers; performance can be affected by non-specific binding.
Vital Signs Monitoring [50,73,74,75,76]	Heart rate, respiration rate, pulse wave.	Doppler radar principle (phase shift) or impedance change via wearable resonant tag.	2–60 GHz for radar; wearable, flexible, or chipless tag form factors.	Adv.: Truly contactless operation (radar); enables integration into textiles/patches. Lim.: Susceptible to motion artifacts; signal strength depends on antenna–skin distance and orientation; challenging for multi-subject environments.
Microfluidic Cell Analysis Sensors [99,100,101,102,103,104,105,106,107,108,109,110,111,112,113]	Cell viability, type, differentiation, intracellular properties (e.g., nucleus).	Broadband dielectric spectroscopy; frequency shift or S-parameter change from cells in microfluidic channel.	Up to 40 GHz for intracellular resolution; integrated microfluidic lab-on-a-chip.	Adv.: Label-free, non-destructive single-cell analysis; can probe sub-cellular structures. Lim.: Complex microfluidic-microwave co-design; lower throughput than flow cytometry; data interpretation relies on accurate bioelectrical models.
Microwave Tomography Systems [137,145,146,147,148,149,150,151,152,153]	Quantitative tissue permittivity map for tumor, stroke, or injury detection.	Inverse scattering: iterative reconstruction of dielectric properties from multi-antenna measurements.	Antenna arrays (1–10 GHz); requires significant computational resources for nonlinear inverse problem.	Adv.: Provides quantitative dielectric property images, useful for tissue characterization. Lim.: Computationally intensive and slow; image reconstruction is ill-posed, requiring regularization; system complexity is high.
Radar-Based Imaging Systems [123,127,135,155,156,157,158,159,160,161,162]	Anatomical localization of tumors (breast, brain), aneurysms, or monitoring of organ movement (heart).	Wideband backscatter analysis; beamforming or time-domain reflectometry to locate dielectric discontinuities.	UWB antenna arrays (1–10 GHz); often portable or wearable system designs.	Adv.: Faster and less computationally demanding than tomography; lower cost and power; good for real-time monitoring. Lim.: Provides qualitative (reflectivity) rather than quantitative images; performance degraded by clutter, multipath, and skin reflections.

## 3. Artificial Intelligence in Medicine

In 1955, John McCarthy and colleagues coined the term Artificial Intelligence (AI), defining it as “the science and engineering of making intelligent machines” [163]. AI, therefore, can be broadly understood as the ability of a machine to imitate intelligent human behavior. Today, AI is increasingly integrated into almost all aspects of daily life, with medicine being one of the fields where its impact is most significant. AI applications in healthcare primarily aim to enhance patient care and improve diagnostic accuracy.

In the medical field, knowledge and experience are the most critical factors for providing effective patient care. The more a physician learns about and treats their patients, the better the quality of care they are able to provide. This process implies that greater experience and access to data lead to more informed, knowledge-based decisions. Such data can be derived from evidence-based medical sources, including textbooks and peer-reviewed research articles, whereas experience is gained through real-world patient outcomes and treatment decisions. However, the human mind is constrained by time and cognitive capacity, limiting their ability to process vast amounts of information efficiently. Thus, AI can play a pivotal role by leveraging extensive medical databases and transforming raw data into actionable knowledge for clinical decision-making and diagnosis [164].

### 3.1. Principal AI Pathways in Medicine

The use of AI in medicine can be classified into two main branches: virtual and physical [165]. The virtual branch is represented by mathematical algorithms that enhance learning through experience. AI has driven and continues to drive significant discoveries in genetics and molecular medicine. For instance, protein–protein interaction algorithms have facilitated the identification of new therapeutic targets by employing adaptive clustering methods [166]. Other virtual applications of AI in medicine include electronic health records, where specific algorithms can be applied to identify individuals with a family history of hereditary diseases, assess high risks of developing chronic conditions, or aid in the detection of rare diseases [167].

AI is also extensively utilized in radiology, particularly due to advances in image recognition and processing technologies. With the increasing availability of digital medical records and enhanced computational power, AI is now at the forefront of medical imaging research. Numerous research groups are actively working on the development of image processing and computer vision algorithms to enable faster diagnoses [168,169], improve pathology visualization [170,171], issue alerts in emergency situations [172], and address healthcare workforce shortages [173]. While these techniques are supported with the different available medical imaging technologies, AI-driven microwave-based medical imaging is a currently burgeoning field with exciting future prospects [174].

Moreover, AI has demonstrated significant utility in oncology. For instance, AI-based diagnostic models have shown superior accuracy compared to human interpretation in the diagnosis and staging of breast cancer [175]. In lung cancer detection, machine learning algorithms have also proved to be more effective than human readings. Findings from [176] suggest that AI can predict lung cancer prognosis with high accuracy, enabling more precise and personalized oncological treatment plans. AI-boosted MWI technologies are also increasingly desirable for the early detection of certain cancers, such as breast cancer [177,178]. AI applications are also prevalent in other medical fields, including cardiology [179], gastroenterology [180], surgery [181], and ophthalmology [182]. While AI-based diagnostics may never achieve absolute certainty, the combination of AI and human medical expertise reliably enhances patient care and clinical decision-making process.

On the other hand, the physical branch of AI involves the use of tangible hardware, medical devices, or even robots, which are becoming increasingly sophisticated and can actively contribute to patient care [183]. One example is the use of emotionally supportive robotic devices (capable of estimating a person’s emotional state) to assist elderly individuals suffering from cognitive decline or limited mobility. In this field, recent research has shown the potential of AI-driven microwave devices for posture and biomechanical monitoring [184], emerging as an excellent complement for these applications.

**Figure 5:**
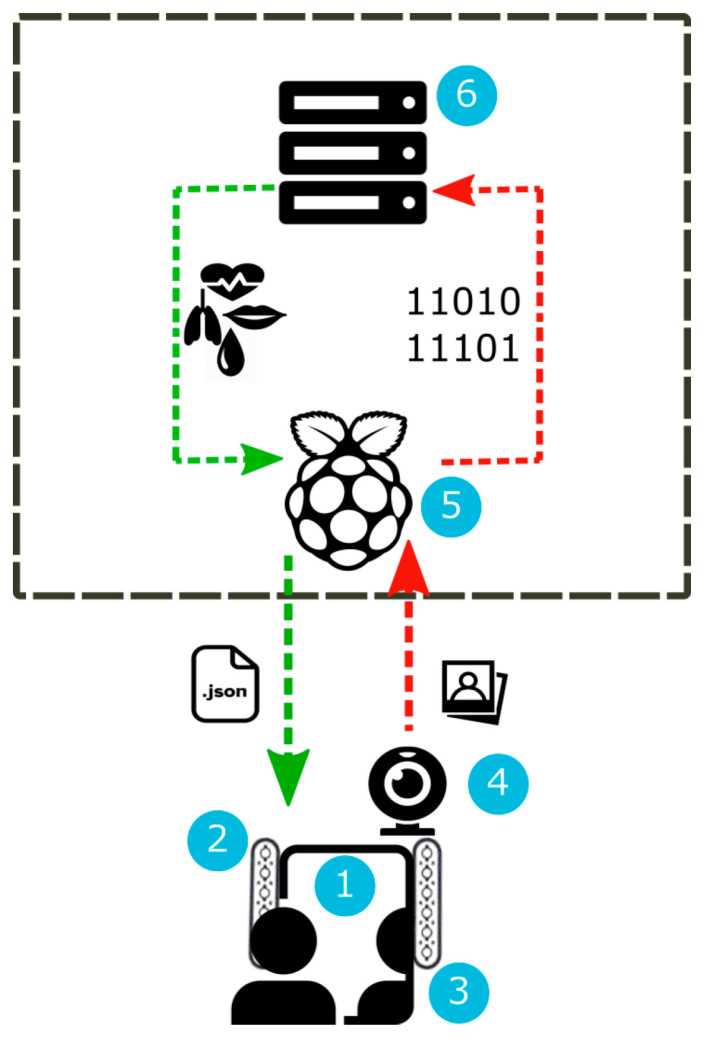
Schematic of the hardware architecture for a cardiovascular monitoring system based on fuzzy logic analysis shown in [185]: (**1**) A see-through mirror; (**2**) a wood frame with two LED strips on the sides for appropriate lighting; (**3**) a monitor behind the mirror used to show vital parameters given by the system after processing; (**4**) a high-definition 1080 p camera for video recording; (**5**) a Raspberry Pi processor board acting as client in a client/server architecture; and (**6**) a computationally powerful desktop computer acting as server, which receives the video frames from the client, processes them, and sends the results back to the client. Reprinted from [185] under CC license.

This category also includes devices designed to improve the quality of life for individuals with neurocognitive disorders, integrating AI-based functionalities. For instance, a real-time cardiovascular monitoring system using video imaging and fuzzy inference rules is described in [185]. The proposed system consists of a transparent mirror equipped with a camera that detects the user’s face. The recorded frames are processed using photoplethysmography to estimate various physiological parameters, which are then analyzed to predict the risk of cardiovascular disease. A schematic of this system is shown in Figure 5. Leveraging their wireless and non-invasive capabilities, microwave sensors are also positioning themselves as good candidates for neurocognitive applications [186]. Furthermore, research making use of mechanical devices has also been published. A system utilizing displacement monitoring devices and machine learning algorithms is presented in [187]. This system comprises devices that track user movement during gait and stimulate muscle nerves via electrical stimulation through electrodes. The process begins with data collection, followed by the training of a recurrent neural network-based model. Ultimately, the trained model predicts user movement during gait and controls the stimulation signal accordingly. In this regard, AI-driven microwave sensors have proved outstanding displacement detection capabilities [188] with interesting application possibilities, such as osteoporosis and bone fracture diagnosis and monitoring [189].

Another application of AI in medicine consists of combining machine learning algorithms with computer vision to develop control and assistance tools for clinical personnel. For instance, in [190], a study presents the development of an algorithm designed to detect surgical hemorrhages during laparoscopic procedures. The model analyzes each frame captured by laparoscopic cameras to classify pixels corresponding to blood. This may be one of the most impactful areas of AI in medicine, leveraging the convolutional neural networks that have revolutionized image analysis. AI models trained on vast datasets can now detect anomalies in medical images with accuracy comparable to or exceeding that of human radiologists. Applications include early detection of cancers [168], automated segmentation of brain tumors [191], and AI-assisted radiology workflows [192], the microwave technology showing enticing prospects in all these fields [193]. The effective merger of these AI capabilities with sensing hardware, particularly for real-time and embedded applications, introduces a distinct set of engineering challenges related to system architecture, computational efficiency, and reliability.

### 3.2. Trends and Future Directions

The rapid advancements in machine learning, particularly in deep learning and deep convolutional neural networks, have strongly fed progress in computer vision, as well as in image analysis and interpretation. Tasks that have traditionally been complex or analytically intractable, such as medical image classification, segmentation, and the identification and recognition of objects of interest, have now become considerably less challenging [194]. These advancements are also reshaping predictive modeling in omics (biology, such as genomics, transcriptomics, proteomics, metabolomics, metagenomics, and phenomics), where the exponential growth of high-throughput sequencing data presents both opportunities and challenges. Traditional machine learning approaches have been partially successful in analyzing omics data but struggle to capture complex relationships for accurate predictions. Deep learning, particularly convolutional neural networks, offers a transformative solution by enabling more effective feature extraction [195]. Methods like DeepInsight convert tabular omics data into image-like representations, allowing convolutional neuronal networks to uncover latent patterns and enhance predictive accuracy [196]. Moreover, the use of transfer learning reduces computational costs while improving performance. However, integrating convolutional neural networks into omics analysis comes with challenges, including model interpretability, data heterogeneity, and scalability [195]. Addressing these issues requires a multidisciplinary effort involving experts from machine learning, bioinformatics, and medical research.

AI-driven predictive models are also reshaping personalized medicine by analyzing patient-specific data to tailor treatments. Deep learning techniques, such as recurrent neural networks and transformer-based architectures, are improving drug discovery [197] and treatment response prediction [198]. This is a particularly active field where microwave technology is enabling notable advancements as for both drug discovery and improved drug delivery [199,200,201].

The implementation of AI in clinical decision support systems has become increasingly important, too, providing real-time assistance to clinicians by analyzing patient data and offering evidence-based recommendations. Natural language processing enables AI models to extract insights from electronic health records, assisting in disease risk assessment [202] and automated patient triage [203]. These advancements have been complemented by AI-powered robotics, electronics, and automation in healthcare, where robotic systems assist in surgical procedures and patient care. Robotic platforms, such as the Da Vinci surgical system, enhance precision in minimally invasive procedures. AI-driven humanoid robots have been developed to address caregiver shortages, assisting the elderly with daily activities and monitoring health conditions. Despite these innovations, AI in medicine faces challenges related to data privacy, model interpretability, and ethical deployment. Ensuring unbiased AI systems and maintaining patient confidentiality remain paramount, frameworks such as “future-AI” have been proposed to guide the trustworthy and ethical implementation of AI in healthcare, emphasizing principles like fairness, transparency, and robustness [204].

AI in medicine is therefore poised for spectacular further growth, with increasing adoption of AI agents to automate administrative tasks and support clinical workflows. Companies are developing AI agents capable of enrolling participants in clinical trials, compiling patient histories, and scheduling appointments, with the goal of reducing physician burnout and improving patient care. In contemporary clinical settings, AI has firmly transitioned from experimental to operational, with widespread deployment across hospitals and health systems. For example, in the United States, approximately two-thirds of radiology departments now utilize AI tools (such as the over 340 FDA-approved applications for detecting tumors, strokes, and hemorrhages) to prioritize urgent cases and expedite diagnostic workflows while maintaining radiologist oversight [205]. Recent pilot studies demonstrate that AI-assisted drafting of radiology reports can significantly reduce reporting time (by nearly 25%), without increasing clinically significant errors, highlighting the tangible efficiency gains of AI integration in routine practice [206]. However, the integration of AI in medicine requires addressing multiple challenges, including model interpretability, data heterogeneity, and scalability (all in the context of societal acceptability and environmental stability, as for any AI system). In this regard, a vast amount of data must be available for trustable AI algorithms to train and use, and microwave devices can potentially become instrumental in data gathering [121].

Beyond hospital operations and diagnostics, AI is increasingly embedded in patient-centered and remote care. Advanced anomaly detection systems like “AI on the Pulse” continuously monitor individuals via wearable sensors, autonomously learning personalized physiological patterns and generating real-time alerts for proactive home-based intervention [207]. Moreover, AI-powered frameworks combining sensor fusion and multivariate analytics have enhanced remote patient monitoring capabilities, enabling early detection of health deterioration, personalized monitoring, and real-time insights, while maintaining data reliability and scalability [208,209]. Again, their wireless, compact, non-invasive capabilities position microwave devices as key components for these systems [121], including applications such as AI-driven microwave-based early detection of Alzheimer’s disease [210].

In summary, AI has evolved from a theoretical concept to a transformative force across virtually all domains of medicine. It now plays a dual role: enhancing clinical decision-making and diagnostic accuracy while simultaneously improving operational efficiency and patient engagement. By supporting physicians with real-time data analysis, predictive modeling, and decision support, AI complements human expertise and contributes to more precise, timely, and personalized care. At the same time, its integration into administrative workflows and patient self-management tools reflects a broader shift toward efficiency and preventive medicine. Although challenges remain regarding transparency, ethics, and clinical validation, the current trajectory confirms that AI is not a future promise but an essential component of modern healthcare practice. For AI to be successfully embedded within next-generation microwave-based diagnostic systems, specific technical hurdles must be addressed, such as optimizing algorithms for edge deployment, ensuring real-time processing, and guaranteeing system safety. These implementation considerations form the focus of the next section. Powered by modern sensing and monitoring technologies, where microwave devices can play a pivotal role, the possibilities of AI in the biomedical realm are limitless.

## 4. Synergistic Combination of Artificial Intelligence and Microwave Technology in Medicine

Microwave technology and AI, as outlined in Section 2 and Section 3, exhibit powerful synergies for biomedical applications. Section 2 showed the immense potential of research on microwave technology for biomedical applications, while Section 3 demonstrated achievable performance gains of off-the-shelf devices and conventional procedures through different AI techniques, particularly machine learning (ML) and deep learning (DL) algorithms. From this perspective, the interest in conducting research involving the simultaneous synergistic combination of advanced microwave devices and AI analysis/processing becomes evident. This has been acknowledged by a growing part of the scientific community in recent years. We now review some of the most relevant works combining these techniques within the biomedical realm.

### 4.1. AI-Enabled Performance Boost

Among the many benefits of AI, two may be singled out as especially relevant for sensing and measurement applications. Firstly, AI can lead to notable increases in the accuracy and robustness of the measurement of certain quantities. For example, advancements have been reported in the measurement of body temperature in wearable or outpatient scenarios (which became especially relevant during and after the COVID-19 pandemic) through linear regression-based ML algorithms with high linearity and low errors [211], including Internet of Things (IoT) capabilities. Microwave radiometry techniques have especially shown performance boost when combined with ML techniques for internal body temperature measurement [212,213], as well as other interesting applications such as skin hydration sensing [214]. Secondly, AI techniques bring new possibilities for the measurement or discernment of new variables, so far blinded or masked. An example is the determination of behavioral, mental, or neural parameters from appropriate processing of physiological variables such as heart rate, pulse rate variability, breath rate, PPG, ECG, EEG, or EDA, among others. For example, while still remaining within a wearable and outpatient setup, mental distress was quantified and classified with decision tree ML techniques with good results [21]. Microwave devices and their novel detection paradigms, propelled by the power of AI, doubtlessly bring new possibilities in this regard [215], such as contactless sleep quality monitoring [216,217,218], for instance.

Another particularly active field of research is solute concentration measurement for biomedical applications, mostly through microwave sensors, allowing the convenient determination of dielectric permittivity properties at these frequencies, as outlined in Section 2.1. Combining the remote or non-invasive capabilities of this technology with the potential of AI leads to intriguing possibilities both for industry-related [219] and biomedical applications [220,221]. In this field, the typically low selectivity is one of the current key challenges [83], to be potentially addressed through AI techniques. Discerning selected variables when multiple changes simultaneously occur in the sample has been proved feasible with analytical approaches provided that the number of varying parameters remains low (i.e., two or three) [61,88,89,222,223,224]. However, as the complexity of the application increases, the analytical extraction of the desired variables becomes unmanageable. At this point, microwave sensors and AI can make a great team.

Exploring this direction, recent works have shown enhanced selective capabilities for multi-solute concentration with microwave sensors and ML techniques, such as principal components regression [225]. These techniques seem particularly relevant for glucose concentration sensing applications, currently a remarkably active field. Microwave sensors combined with AI techniques have been tested in human trials, involving DL algorithms such as Long Short-Term Memory (LSTM) [226,227] or Super-Resolution Generative Adversarial Network (SRGAN) [228], as well as ML algorithms such as Light Gradient-Boosting Machine (LightGBM) [229], Random Forests Regressor (RF) [230] or MultiLayer Perceptron (MLP) [231]. All these works have shown notably enhanced glucose measurement capabilities with high specificity for glucose level challenges, proving their merit in addressing the selectivity challenge. As a limitation, the human trials in these studies have, to date, involved too few volunteers (less than 10 in all cases), which is not enough for rigorous validation.

Other efforts have led to proposed AI techniques for resolution enhancement of microwave sensors in microfluidic contexts, with DL algorithms such as CycleGAN [232], Convolutional Neural Networks (CNN) [233] or Artificial Neural Networks (ANN) [234], or ML algorithms such as General Regression Neural Network (GRNN) [235], all of which have notably improved sensor accuracies. Techniques other than microwaves, such as optical sensors, have also been combined with AI processing for glucose sensing, with ML algorithms such as Support Vector Machine (SVM) [236], although less frequently. Finally, microwave biosensors have been combined with AI techniques for other types of sensing, such as oedema severity classification through ML SVM processing [237] or bacteria detection in body fluids by means of ML RF [220].

### 4.2. AI-Driven Optimized Design

As shown, AI has proved to be broadly effective for computing applications in complex environments or scenarios. This is clearly the case with free space propagation, reflection, and scattering of electromagnetic waves through different media, which is the basis for MWI and antenna-related applications in general [238]. The burgeoning research activity at the intersection of AI, antenna design, and electromagnetic wave propagation methodologies for biosensing applications is expected to continue into the foreseeable future [239,240].

Importantly, AI techniques can not only boost system performance in many applications, but also help in optimizing the hardware design itself. For instance, automated algorithms based on Boolean transformations, evolutionary algorithms, and gradient-based optimizers were proposed for unsupervised design of highly sensitive microwave resonant sensors [241], or optimization algorithms were combined with DL techniques based on a U-Net architecture for optimizing the design of the pattern of the antennas in a microwave imaging system [242], to name a few. Similar techniques have also been studied for other biosensing scenarios. Ultra-Wideband (UWB) antennas were used to monitor vital signs, such as heart rate, respiratory rate, and even lung water level by means of several ML algorithms [243], and radar apparatuses were powered with DL processing for respiratory monitoring [244], to name a few. These advancements also find application in non-biomedical industries, such as the control of food and beverage products. Here, the ML SVM technique has been deployed in conjunction with a tunnel-shaped antenna assembly for a conveyor belt monitoring system [245], for example.

### 4.3. Impact of AI in Medical Imaging

Within the field of medicine, MWI systems are particularly well poised for improvement with AI techniques. AI addresses core limitations of MWI across two main fronts: image reconstruction and image analysis.

**Figure 6:**
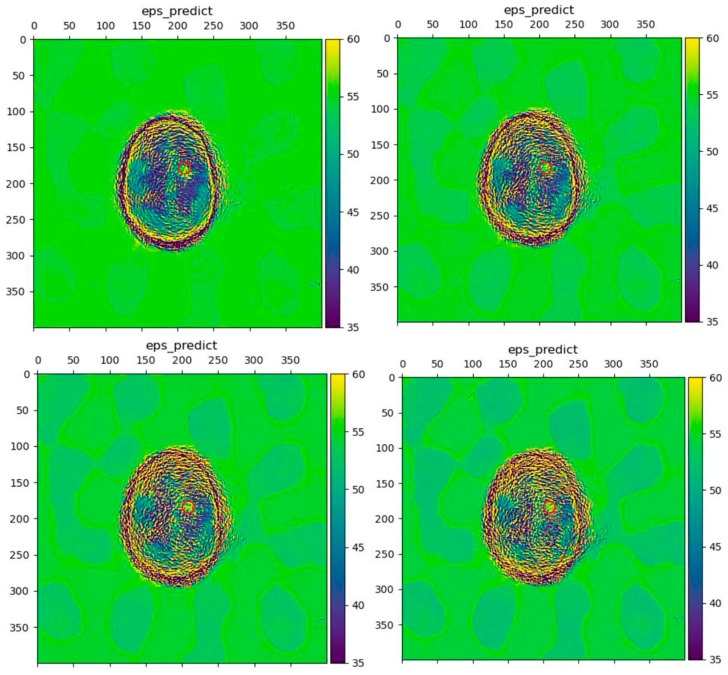
Reconstructed brain-stroke screening images under different intensities of noise (4%, 6%, 8%, and 10% in (**top left**), (**top right**), (**bottom left**), and (**bottom right**), respectively) according to the technique in [150], where the red circle shows the true position. Reprinted from [150] under CC license.

In image reconstruction, AI mitigates the inherent computational complexity and ill-posed nature of inverse scattering problems. Traditional iterative algorithms for microwave tomography are computationally intensive and sensitive to noise and model inaccuracies. DL approaches, particularly CNNs and physics-informed neural networks (PINNs), learn direct mappings from scattered field data to dielectric property maps or to initial estimates that significantly accelerate convergence [150,246]. These methods can implicitly regularize the solution, enhancing robustness to measurement noise and reducing the need for extensive calibration, as demonstrated in noise-resilient stroke imaging (see Figure 6).

In image analysis, AI excels at the automated detection and classification of pathologies within reconstructed MWI data. Here, the challenge shifts from solving a physics-based inverse problem to a pattern recognition task within often low-contrast or noisy images. DL architectures, such as U-Net for segmentation [246,247] and CNNs for classification [248,249,250], are trained to identify the subtle, complex signatures of malignancies (e.g., in breast tissue [251]) or anomalies (e.g., strokes, tumors) that may be indistinct to human observers. This capability is crucial for translating MWI into a practical diagnostic tool, providing quantitative, user-independent assessments.

As shown, the scientific community has reached a general consensus on the suitability of DL in MWI, the vast majority of reports being based on these techniques. The prevailing research trajectory involves moving from purely data-driven models to hybrid approaches that incorporate electromagnetic physical constraints, aiming to improve generalizability, reduce data requirements, and provide physically plausible outputs, which remain a key concern for clinical trustworthiness.

### 4.4. Implementation Architectures and System-Level Challenges

The integration of AI into functional microwave-based medical devices introduces critical engineering decisions and challenges beyond algorithmic performance. The choice of implementation architecture, spanning the continuum from cloud-based processing to fully embedded edge AI, directly impacts system utility, safety, and clinical viability.

A primary architectural decision involves the computing paradigm. For data-intensive tasks like high-resolution microwave image reconstruction or training of complex models, cloud-based processing offers virtually unlimited computational power and ease of updates [205,252]. However, it necessitates constant, high-bandwidth connectivity, introduces latency, and raises concerns about data privacy and transmission security [253]. Conversely, for real-time, continuous monitoring applications (e.g., vital sign tracking, fall detection, and glucose alerting), embedded edge AI is preferable. Here, lightweight models are deployed directly on the sensor hardware or a local gateway, enabling immediate inference, operation without connectivity, and enhanced user privacy [207,254]. The development of TinyML and model optimization techniques (e.g., pruning and quantization) is crucial to fit performant models into the constrained memory and power budgets of wearable microwave sensors.

Meeting the real-time processing demands of medical monitoring, such as detecting cardiac arrhythmias or apnea events with sub-second latency, requires careful co-design of the AI model and the microwave sensor system. This involves trading off model complexity (accuracy) for inference speed and power efficiency on edge hardware, which must also be designed for reliable operation in ambulatory environments, managing issues such as motion artifacts, thermal management, and battery life [208].

Beyond performance, the deployment of AI in medical systems mandates exceptional reliability and safety. Models must be robust to noisy, out-of-distribution, or adversarial input data. Explainability and interpretability of AI decisions remain a significant hurdle for clinical adoption and trust [204,255]. Fail-safe mechanisms are essential, and systems should be designed to default to a safe state or provide clear uncertainty estimates when AI confidence is low, rather than providing a potentially erroneous output. Establishing rigorous validation frameworks and standards for AI-enabled medical devices is an ongoing priority for regulatory bodies [204].

In summary, transitioning from proof-of-concept AI algorithms to deployed clinical systems requires solving a multi-faceted set of implementation challenges. Future work must focus on developing resource-efficient AI models tailored for microwave hardware, creating robust and interpretable systems, and establishing clear regulatory pathways for these convergent technologies.

## 5. Challenges, Limitations, and Future Directions at the Microwave-AI Frontier

The convergence of microwave sensing and artificial intelligence, as surveyed in this manuscript, holds immense promise for transforming biomedical monitoring and imaging. However, translating this promise into widespread clinical practice requires a clear-eyed assessment of the persistent challenges and limitations that span technical, algorithmic, and practical domains. A comparative evaluation of the primary technology paradigms discussed in this review is presented in Table 2, summarizing their key advantages, inherent limitations, and the corresponding role of AI. This section subsequently synthesizes these cross-cutting hurdles and outlines critical pathways for future research.

**Table 2:** Comparative evaluation of microwave–AI integration paradigms for biomedical applications.

Technology Paradigm (Primary Application)	Key Strengths/Advantages	Principal Limitations/ Challenges	Critical AI Integration Role and Future Needs
Planar Resonator Sensors (Biomarker Monitoring)	Non-invasive capability; low-cost, planar fabrication; potential for wearability.	Inherent low selectivity; susceptible to confounding variables (temperature, other solutes, etc.).	Role: Multi-parameter analysis for selectivity; calibration drift correction. Need: Large, clinically validated datasets; explainable models for trust.
Wearable/Radar-based Vital Sign Monitors (Heart Rate, Respiration)	Truly contactless operation; enables continuous, long-term monitoring.	Sensitive to motion artifacts; limited to superficial/significant physiological signals.	Role: Signal denoising; activity context identification; anomaly detection. Need: Robust, lightweight models for edge processing; privacy-by-design architectures.
Microfluidic Cell Sensors (Single-Cell Analysis)	Label-free, non-destructive; can probe sub-cellular dielectric properties.	Low throughput; complex microfluidic integration; requires biophysical models for interpretation.	Role: Automated cell classification; real-time analysis of dielectric spectra. Need: Standardized data formats; hybrid models combining physics and AI.
Microwave Tomography (Tumor/Stroke Imaging)	Provides quantitative permittivity maps; excellent for tissue characterization.	Computationally intensive (nonlinear inverse problem); slow imaging speed; complex system setup.	Role: Accelerating image reconstruction; improving solution uniqueness. Need: Physics-informed neural networks (PINNs); efficient inverse solvers.
Radar-based Imaging (Tumor Localization, Organ Motion)	Faster imaging speed; lower system cost and complexity; portable designs feasible.	Qualitative images (scatterer location vs. permittivity); clutter and multipath interference.	Role: Advanced beamforming; clutter rejection; automatic anomaly detection. Need: AI for improved image qualitative accuracy; fusion with other modalities.

### 5.1. Technical and Physical Limitations of Microwave Sensing

The fundamental physics of microwave-tissue interaction presents inherent trade-offs. While advantageous for non-ionizing, deep-tissue penetration, it also leads to limited spatial resolution compared to modalities like X-ray or MRI. Furthermore, the dielectric properties of biological tissues are non-specific; changes due to a target biomarker (e.g., glucose) can be masked or mimicked by confounding variables such as temperature fluctuations, hydration level, or the presence of other solutes [83,85,86]. This creates a fundamental selectivity challenge, especially for continuous monitoring applications. At the system level, designing hardware that is simultaneously sensitive, miniaturized, power-efficient, and robust to motion artifacts or environmental interference remains a significant engineering obstacle. While strategies like coupled resonators [77,78,79] and lossy sensors [80,81,82] enhance sensitivity, integrating them into stable, wearable form factors is an ongoing effort.

### 5.2. Data and Algorithmic Hurdles for AI Integration

The effectiveness of AI is contingent on the quality and scale of data. A primary limitation across the field is the scarcity of large, curated, and clinically validated datasets specific to microwave-based biosensing and imaging. Many promising studies, particularly in non-invasive glucose monitoring, are validated on prohibitively small volunteer cohorts [226,227,231], limiting statistical power and generalizability. Future work must prioritize the creation of open, benchmark datasets.

Algorithmically, the predominance of complex deep learning models exacerbates issues of interpretability and explainability. Deploying “black-box” models in safety-critical medical applications is problematic. Techniques such as SHAP (SHapley Additive exPlanations) analysis [256] should be leveraged to unravel the model decisions and build clinician trust. Furthermore, models must be designed for efficiency, utilizing techniques like pruning and quantization to enable real-time inference on edge hardware with limited power and memory, a necessity for wearable and point-of-care devices [207].

Finally, algorithmic bias and data privacy are paramount concerns within the Internet of Medical Things (IoMT) framework. Ensuring that models are trained on diverse datasets and deploying privacy-preserving techniques like federated learning will be essential for equitable and secure AI deployment [253,255,257].

### 5.3. Clinical Translation and Practical Deployment Challenges

Beyond laboratory performance, successful translation requires overcoming a distinct set of barriers [258]. Clinically, the ultimate benchmark consists in improving patient outcomes. This demands rigorous, large-scale clinical trials that move beyond technical feasibility to demonstrate diagnostic accuracy, cost-effectiveness, and workflow integration compared to standard of care.

The choice of implementation architecture (viz. cloud versus edge computing) presents practical trade-offs. Cloud-based processing facilitates complex model updates and heavy computation but introduces latency, connectivity dependence, and data security risks [254]. Edge AI enables real-time, private analysis but faces strict hardware constraints [208]. Hybrid models may offer a viable path forward.

Regulatory pathways for these convergent AI-enabled medical devices are still evolving. Agencies require robust evidence of safety, efficacy, and algorithmic transparency [204]. Concurrently, addressing ethical frameworks, ensuring equitable access to avoid exacerbating health disparities, and designing fail-safe operational protocols are non-technical but critical prerequisites for clinical adoption [255,257].

### 5.4. Concluding Synthesis and Emerging Pathways

The synergistic combination of microwave technology and AI is not merely an incremental improvement but a paradigm shift towards intelligent, pervasive, and personalized healthcare monitoring. As this review illustrates, progress has been rapid across multiple fronts: from AI-enhanced sensor selectivity [225,226,227,228,229,230,231] and optimized hardware design [241,242] to revolutionary advances in medical image reconstruction [246,247,248,249,250,251].

The path forward is inherently multidisciplinary. Future research must tightly couple advances in microwave engineering (developing more selective and robust sensors), data science (creating explainable and efficient algorithms), and clinical medicine (designing outcome-driven validation studies). Prioritizing the creation of shared datasets, developing standards for performance reporting, and fostering collaboration across engineering, computer science, and clinical domains will be crucial. By systematically addressing the technical limitations, data scarcity, algorithmic opacity, and clinical translation barriers outlined here, the field can mature from producing compelling proofs-of-concept to delivering trustworthy, accessible, and life-saving diagnostic tools that fulfill the transformative potential of this powerful technological convergence.

## 6. Conclusions and Perspectives

This article has analyzed the burgeoning convergence of microwave sensing technology and artificial intelligence for biomedical monitoring and imaging. The surveyed literature reveals intense, parallel advancements in both fields, which are now merging into a synergistic and transformative research frontier. Significant progress is evident across the stack: from the development of sensitive, miniaturized microwave hardware to the application of sophisticated AI algorithms for signal interpretation, hardware optimization, and image reconstruction. Looking ahead, this convergence is poised to enable a new generation of medical technologies, including AI-optimized microwave biosensors for continuous biomarker tracking, predictive personal health monitoring systems, and highly accessible, AI-enhanced diagnostic imaging devices.

However, as detailed in the discussion of challenges, the path from compelling research to clinical adoption is non-trivial. Realizing this future requires concerted, multidisciplinary efforts to overcome fundamental technical hurdles (e.g., selectivity and miniaturization), critical data and algorithmic bottlenecks (e.g., dataset scarcity, and model explainability), and pragmatic translation barriers (e.g., clinical validation, regulatory pathways, and ethical deployment). Success will hinge on the tight co-design of microwave engineering, data science, and clinical medicine. Ultimately, the fusion of microwave technology and AI represents more than an incremental improvement: it heralds a paradigm shift toward intelligent, pervasive, and personalized healthcare. By systematically addressing the outlined challenges, the field can transition from producing innovative proofs-of-concept to delivering robust, trustworthy, and equitable diagnostic tools, thereby unlocking the full potential of this powerful synergy to improve patient outcomes and redefine biomedical monitoring.

## Data Availability

No new data were created or analyzed in this study.
